# Developing a framework for identifying risk factors and estimating direct economic disease burden attributable to healthcare-associated infections: a case study of a Chinese Tuberculosis hospital

**DOI:** 10.1186/s41256-024-00375-w

**Published:** 2024-09-09

**Authors:** Nili Ren, Xinliang Liu, Yi Luo, Guofei Li, Ying Huang, Desheng Ji, Cheng Peng, Jing Sun, Hao Li

**Affiliations:** 1 Wuhan Pulmonary Hospital/Wuhan Institute for Tuberculosis Control, No28 Baofengyilu Road, Wuhan, 430030 China; 2 School of Public Health/Global Health Institute, Wuhan University, No. 115 Donghu Road, Wuhan, 430071 China; 3 Division of Population Health, Health Services Research and Primary Care, School of Health Sciences, Faculty of Biology, Medicine and Health, The University of Manchester, Manchester Academic Health Sciences Centre, Oxford Road, Manchester, M13 9PL UK; 4 School of Health Policy and Management, Dongcheng District, Chinese Academy of Medical Sciences and Peking Union Medical College, No. 9 Dongdan Santiao, Beijing, 100730 China

**Keywords:** Healthcare-associated infections, Risk factors, Direct economic disease burden, Case study, Framework

## Abstract

Healthcare-associated infections (HAIs) represent a major global health burden, which necessitate effective frameworks to identify potential risk factors and estimate the corresponding direct economic disease burden. In this article, we proposed a framework designed to address these needs through a case study conducted in a Tuberculosis (TB) hospital in Hubei Province, China, using data from 2018 to 2019. A comprehensive multistep procedure was developed, including ethical application, participant inclusion, risk factor identification, and direct economic disease burden estimation. In the case study, ethical approval was obtained, and patient data were anonymized to ensure privacy. All TB hospitalized patients over the study period were included and classified into groups with and without HAIs after screening the inclusion and exclusion criteria. Key risk factors, including gender, age, and invasive procedure were identified through univariate and multivariate analyses. Then, propensity score matching was employed to select the balanced groups with similar characteristics. Comparisons of medical expenditures (total medical expenditure, medicine expenditure, and antibiotics expenditure) and hospitalization days between the balanced groups were calculated as the additional direct economic disease burden measures caused by HAIs. This framework can serve as a tool for not only hospital management and policy-making, but also implementation of targeted infection prevention and control measures. Moreover, it has the potential to be applied in various healthcare settings at local, regional, national, and international levels to identify high-risk areas, optimize resource allocation, and improve hospital management and governance, as well as inter-organizational learning. Challenges to implement the framework are also raised, such as data quality, regulatory compliance, considerations on unique nature of communicable diseases and other diseases, and training need for professionals.

**Supplementary Information:**

The online version contains supplementary material available at 10.1186/s41256-024-00375-w.

## Introduction

Healthcare-associated infections (HAIs) pose a major global health burden. The World Health Organization (WHO) reported that in 2022, globally in acute-care hospitals, about 7 out of every 100 hospitalized patients in high-income countries (HICs) and 15 out of every 100 hospitalized patients in low- and middle-income countries (LMICs) obtained at least one HAI, and an average of 1 in every 10 hospitalized patients with HAIs died [1]. HAIs also lead to the increased occurrence of antimicrobial resistance (AMR)—another major global health issue [2]. However, from the perspective of the economic disease burden attributable to HAIs, the updated evidence is scant, especially in settings with limited resources. A framework is required to estimate the direct economic burden attributable to HAIs at minimum level-in individual hospitals, providing empirical evidence and support targeted interventions. In response to the global challenge of HAIs, the WHO announced its first-ever global strategy on infection and prevention control (IPC) at the 76th World Health Assembly in 2023, and an associated global action plan and monitoring framework will be completed by 2024 [3]. Effective and tailored IPC measures are formulated based on identifying the risk factors associated with HAIs within hospitals. The set of risk factors associated with HAIs could be different in different hospitals. Therefore, a general framework should be developed to identify the risk factors associated with HAIs unique to each hospital setting.

The framework on estimating the direct economic disease burden attributable to HAIs was developed before by our team and applied within general hospitals in China and Nepal [4–6]. However, it has yet to be used in specialized hospitals, such as those focusing on Tuberculosis (TB). Specialized hospitals, particularly TB hospitals, face unique challenges that general hospitals may not encounter, such as managing patients with complex and prolonged treatment needs, making them more vulnerable to HAIs. Applying the framework in such setting is important to address these specific challenges and refine infection control strategies. While the framework itself remains consistent, its application in TB hospitals allow for the adaptation of infection control measures tailored to the unique needs of TB patients. The risk of HAIs is further compounded by the nature of TB itself-a deadly respiratory infectious disease, which remains a significant global health issue. According to the latest estimates reported by the WHO in 2023, around 10.6 million population were estimated to be infected with TB worldwide, and 1.3 million of them died in 2022 [7]. This highlights the ongoing need for effective management and control of TB, making it a relevant focus for investigating HAIs. Particularly, the South-East Asia Region had the highest number of population infected with TB at 4.85 million, followed by the African Region at 2.48 million [7]. These figures emphasize the global burden of TB and the necessity for targeted interventions in high-prevalence areas. The high incidence and the complex nature of TB treatment environment can increase the vulnerability of patients to HAIs, thus escalating the economic and health burden on healthcare systems. TB treatment often involves prolonged hospital stays [8], intensive antibiotics use [9], and invasive procedures [10], all of which elevate the risk of HAIs. Implementing the framework in specialized hospitals helps gather data that are more relevant to these settings, providing insights into the specific economic burden of HAIs in TB hospitals and offering a clearer picture of the financial impact and resource requirements for effectively managing HAIs in specialized care environments.

To address above situation, a framework was developed by our team based on the case study conducted in a Chinese TB hospital to analyze the potential risk factors and estimate the direct economic burden attributable to HAIs. Furthermore, this framework aims to provide healthcare stakeholders with a tool for implementing effective IPC measures and evaluating the financial impact of HAIs. The Chinese TB hospital is located in Hubei Province of China, which is a tertiary pulmonary and tuberculosis control hospital that focuses on the prevention and control of TB, clinical diagnosis and treatment of pulmonary diseases, and medical/health education and research. This TB hospital specifically handles complex cases of TB, including multi-drug-resistant TB (MDR-TB), which require prolonged treatment and intensive care. It is a specialty hospital at a city level. Compared to other TB hospitals at a city level in Hubei Province, it is the only tertiary hospital, while others are secondary hospitals. Table 1 shows the information of the operational and treatment efficacy about the hospital from 2018 to 2019. The number of beds and average hospitalization days remained stable at 406 and 9.73 days, respectively. The mortality rate of hospitalized patients increased from 0.45 to 0.72%, and the number of outpatient and emergency patients rose significantly from 113,819 to 129,905 in this TB hospital. The annual HAIs prevalence decreased from 0.53 to 0.34%.

**Table 1:** General information of the Chinese TB hospital

Year	2018	2019
Number of beds	406	406
Average hospitalization days	9.73	9.73
Total mortality of hospitalized patients (%)	0.45	0.72
Number of discharged patients	16,445	16,649
Number of outpatient and emergency patients	113,819	129,905
Annual HAIs prevalence (%)	0.53	0.34

## Framework development

**Fig. 1:**
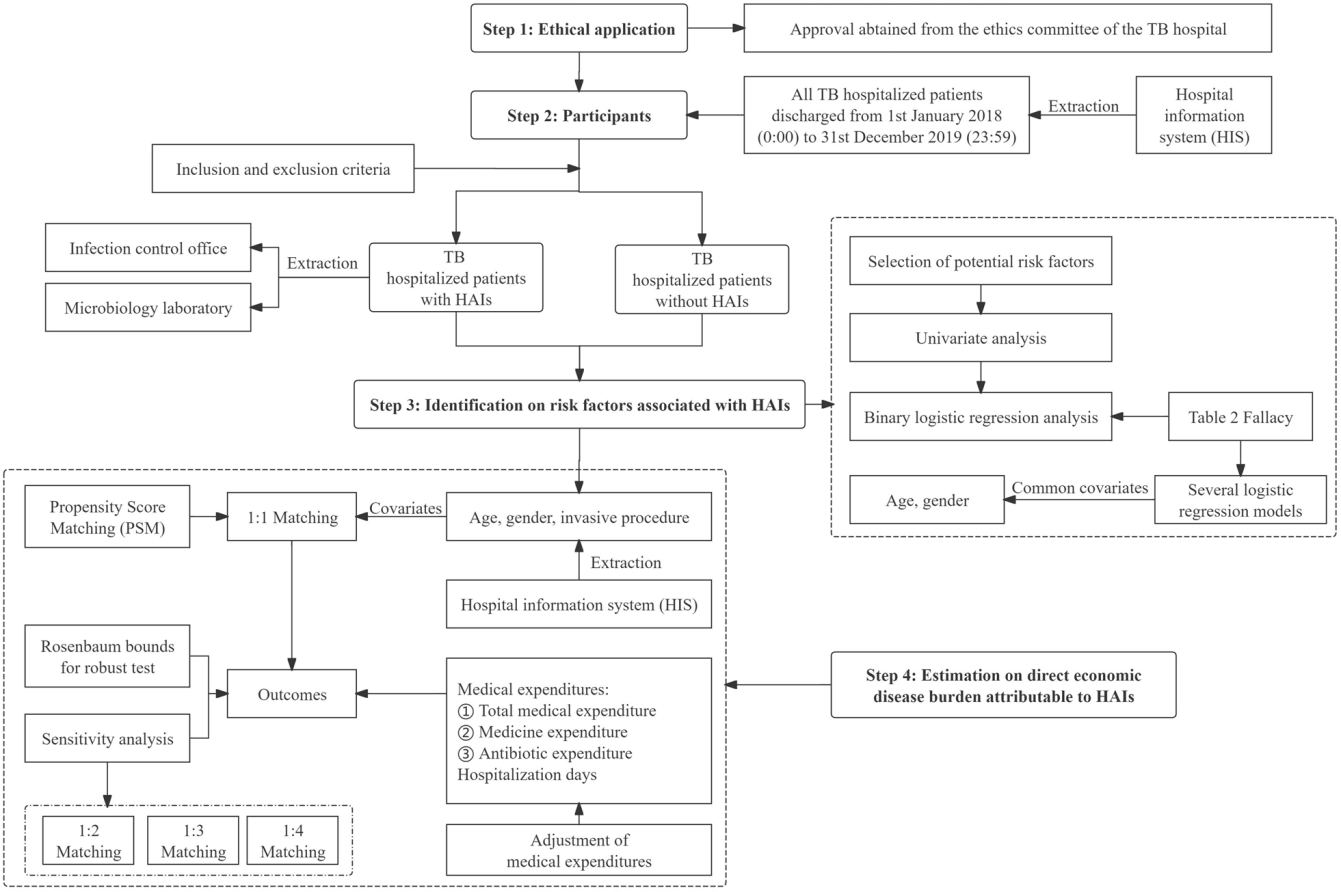
Flow chart of the framework on identification of risk factors and estimation of the direct economic disease burden attributable to HAIs

Figure 1 presents that a framework analyzes the impact of HAIs within a hospital setting, which includes four steps. The first step is an ethical application, ensuring that all data collection and analysis adhere to the highest standards of research ethics. The first step secures the approval of relevant ethics committees and establishes a foundation of trust and legality for research. Then, the second step is the inclusion of participants, which involves a detailed screening process to select all eligible hospitalized patients based on specific criteria. This selection process ensures that the data extracted are relevant and robust, providing a solid base for further analysis. The third step is that the framework identifies risk factors associated with HAIs, which involves a thorough analysis of patient data to ascertain factors that may increase the likelihood of HAIs, such as the length of hospitalization, the use of invasive procedure, or the presence of comorbid conditions. Understanding these risk factors is pivotal for developing targeted strategies to reduce the incidence of HAIs. Finally, the fourth step is the estimation of the direct economic disease burden attributable to HAIs. By identifying the significant risk factors in the third step, we could accurately calculate the additional medical expenditures and hospitalization days, using these identified risk factors as covariates. The economic burden analysis helps to quantify the financial impact of HAIs, highlighting the economic incentives for hospitals to invest in effective IPC measures. Overall, the framework employs a retrospective cross-sectional study design, allowing for the analysis of data from previously hospitalized patients. This approach is advantageous as it provides a snapshot of hospital performance over a specific period, enabling hospital managers and policy decision-makers to implement evidence-based improvements in patient care and IPC measures. This comprehensive and methodical approach ensures that every aspect of the impact of HAIs is captured and addressed, including from ethical considerations to risk factor analysis and economic burden analysis.

### Step 1: ethical application

Before extracting data from hospitals, it is essential to secure approval from the hospitals’ ethics committees, particularly when the data involve personal information about hospitalized patients. This case study obtained the approval from the TB hospital’s ethic committee (Wuhan Pulmonary Ethic Committee (2021) 28). To ensure anonymity, original hospital numbers were replaced with unique identifiers created by a staff member from the Department of Medical Records. Personal information pertaining to the hospitalized TB patients was omitted during data extraction from the hospital information systems (HIS). As a result, there was no need for informed or verbal consent from the TB hospitalized patients.

### Step 2: inclusion of participants

**Fig. 2:**
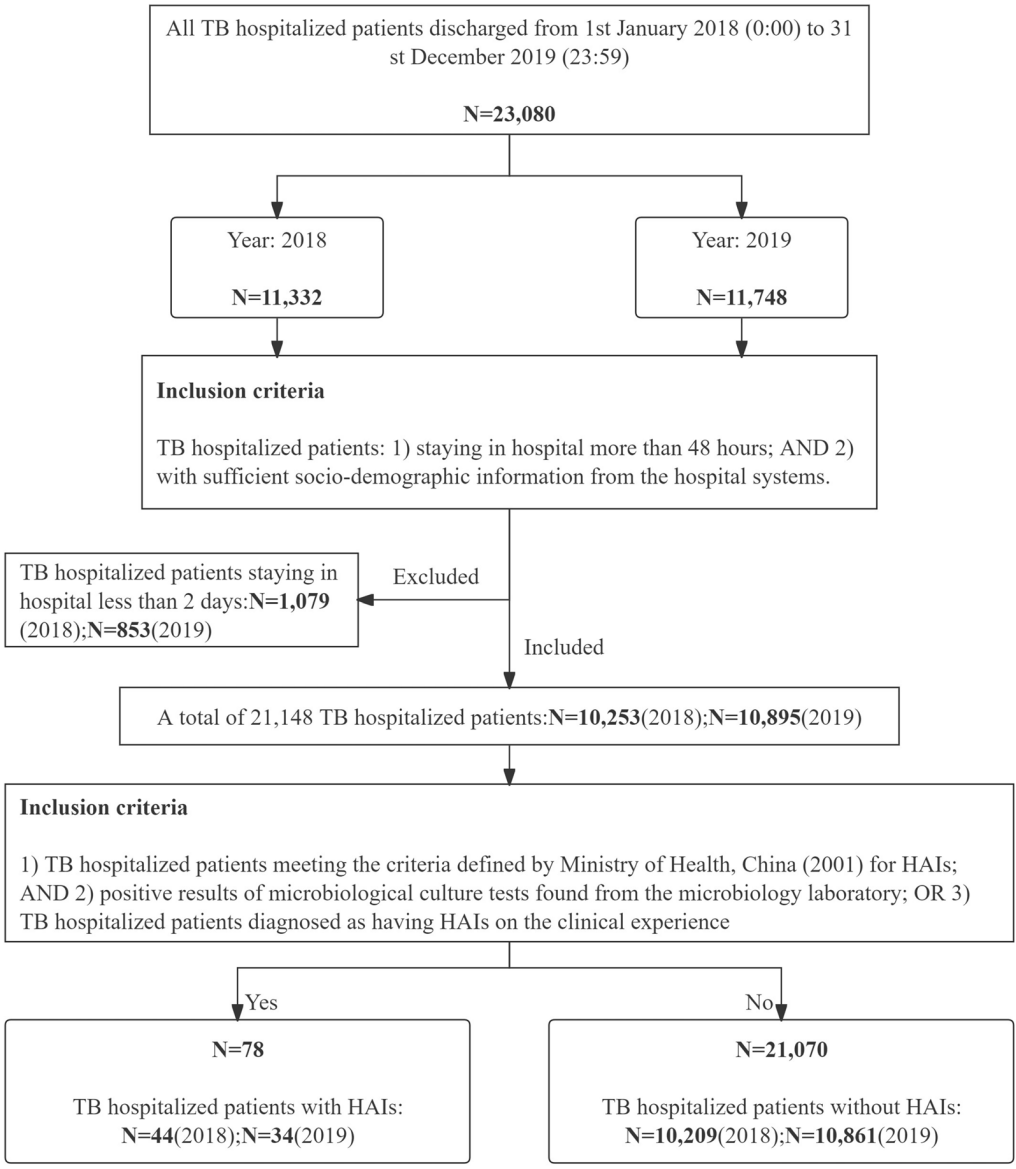
Flow chart of the included participants from the Chinese TB hospital in 2018 and 2019

This phrase primarily involves the inclusion of all hospitalized patients, followed by their classification into groups with and without HAIs for subsequent analysis of risk factors and economic burden. The specific steps taken in this TB hospital were as follows, with Fig. 2 illustrating the entire participant inclusion flowchart:

1. All TB hospitalized patients who were discharged from 0:00 1st January 2018 to 23:59 31st December 2019 were included. TB hospitalized patients information was retrieved from the HIS. Due to the COVID-19 pandemic, the local government placed strict restrictions on data sharing among local hospitals, resulting in inaccessible data from 2020 to 2022. As demonstrated in Fig. 2, a total of 23,080 TB hospitalized patients were included during the study periods, with 11,332 patients in 2018 and 11,748 in 2019.
2. Those TB hospitalized patients staying in hospital less than 48 h were excluded, since the criteria for HAIs require a minimum hospital stay of more than two days [11]. After applying this exclusion criterion, the total number of included TB hospitalized patients was reduced to 21,148.
3. The remaining TB hospitalized patients were then categorized based on whether they had acquired HAIs, according to the inclusion and exclusion criteria detailed in Additional file 1: Table S1. Following this categorization, 78 TB hospitalized patients were identified with HAIs, while 21,070 TB hospitalized patients did not have HAIs.

### Step 3: identification on risk factors associated with HAIs

The risk factors analysis on association with HAIs among hospitalized patients typically involves selecting potential risk factors, conducting univariate analysis, and performing logistic regression analysis. Here are the specific steps taken at this TB hospital:

1. Our research team has conducted a systematic review and meta-analysis to identify risk factors associated with HAIs among TB hospitalized patients in China [12]. This systematic review concluded a list of significant risk factors, including age older than 60 years, presence of complications, diabetes mellitus, invasive procedure, longer than 15 hospitalization days, secondary TB, smoking, presence of underlying disease, and use of antibiotics [12]. This comprehensive review provided a robust foundation for identifying potential risk factors in the TB hospital to collect data. Considering the data availability from this TB hospital, gender, age older than 60 years, diabetes mellitus, invasive procedure, more than 15 hospitalization days, presence of underlying disease, and used of antibiotics were selected as potential risk factors in this case study. Descriptions and assigned values of the selected potential risk factors are listed in Additional file 1: Table S2.
2. A univariate analysis was conducted to examine the association between the selected risk factors and HAIs among the TB hospitalized patients. The chi-squire test was used for those categorical variables with expected frequencies above five, while the Fisher exact probability test was used for those with frequencies below five. The codes for conducting the univariate analysis can be found in Additional file 2. Table 2 demonstrates that gender, longer than 15 hospitalization days, invasive procedure, and the use of antibiotics were consistent risk factors, each showing a statistically significant association with HAIs among TB hospitalized patients in both 2018 and 2019 (*P* < 0.05).
3. A binary logistic regression model was applied to investigate the severity of potential risk factors associated with HAIs among TB hospitalized patients. To avoid ‘Table 2 Fallacy’ where multiple adjusted odds ratios (aOR) derived from a single logistic regression model misinterpret the impact of primary risk factors due to covariate heterogeneity [13, 14], several separate binary logistic regression models were conducted by adjusting gender and age, as both are general socio-demographic characteristics and are common and likely to be prognostic. The codes for this binary logistic regression analysis are available the in Additional file 2. Table 3 shows that consistently, the significant risk factors associated with HAIs were invasive procedure (aOR 7.41 in 2018; 4.29 in 2019), longer than 15 hospitalization days (aOR 13.15 in 2018; 39.76 in 2019), and use of antibiotics (aOR 8.99 in 2018; 33.46 in 2019) (*P* < 0.05).

**Table 2:** Univariate analyses on potential risk factors associated with HAIs in 2018 and 2019

Year	Potential risk factors		TB hospitalized patients with HAIs	TB hospitalized patients without HAIs	*χ*^*2*^	*P*
			N (%)			
2018	Gender	Male	40 (90.91)	6413 (62.82)	14.82	< 0.01*
		Female	4 (9.09)	3796 (37.18)		
	Age	> 60 years	18 (40.91)	2512 (24.61)	6.26	0.012*
		≤ 60 years	26 (59.09)	7697 (75.39)		
	Invasive procedure	Yes	12 (27.27)	492 (4.82)	–	< 0.01*
		No	32 (72.73)	9717 (95.18)		
	Length of hospitalization	> 15 days	32 (72.73)	1639 (16.05)	103.15	< 0.01*
		≤ 15 days	12 (27.27)	8570 (83.95)		
	Diabetes mellitus	Yes	5 (11.36)	1293 (12.67)	0.07	0.796
		No	39 (88.64)	8916 (87.33)		
	Underlying disease	Yes	11 (25.00)	2272 (22.25)	0.19	0.662
		No	33 (75.00)	7937 (77.75)		
	Use of antibiotics	Yes	40 (90.91)	5095 (49.91)	29.46	< 0.01*
		No	4 (9.09)	5114 (50.09)		
2019	Gender	Male	27 (79.41)	6760 (62.24)	4.26	0.039*
		Female	7 (20.59)	4101 (37.76)		
	Age	> 60 years	13 (38.24)	2685 (24.72)	3.32	0.068
		≤ 60 years	21 (61.76)	8176 (75.28)		
	Invasive procedure	Yes	7 (20.59)	659 (6.07)	–	0.004*
		No	27 (79.41)	10,202 (93.93)		
	Length of hospitalization	> 15 days	30 (88.24)	1660 (15.28)	137.64	< 0.01*
		≤ 15 days	4 (11.76)	9201 (84.72)		
	Diabetes mellitus	Yes	7 (20.59)	1743 (16.05)	0.52	0.472
		No	27 (79.41)	9118 (83.95)		
	Underlying disease	Yes	7(20.59)	2580 (23.75)	0.19	0.665
		No	27 (79.41)	8281 (76.25)		
	Use of antibiotics	Yes	33 (97.06)	5180 (47.69)	33.10	< 0.01*
		No	1 (2.94)	5681 (52.31)		

* means a statistical significance (*P*<0.05)

**Table 3:** Logistic regression analyses on potential risk factors associated with HAIs in 2018 and 2019

Year	Potential risk factors	Reference	aOR	95%CI	Z	*P*
2018	Invasive procedure	No	7.41	[3.78–14.54]	5.83	< 0.01*
	Length of hospitalization	≤ 15 days	13.15	[6.75–25.61]	7.57	< 0.01*
	Diabetes mellitus	No	0.66	[0.26–1.70]	− 0.85	0.395
	Underlying disease	No	0.78	[0.38–1.63]	− 0.66	0.512
	Use of antibiotics	No	8.99	[3.21–25.18]	4.18	< 0.01*
2019	Invasive procedure	No	4.29	[1.85–9.93]	3.40	0.001*
	Length of hospitalization	≤ 15 days	39.76	[13.98–113.08]	6.90	< 0.01*
	Diabetes mellitus	No	1.08	[0.46–2.52]	0.17	0.864
	Underlying disease	No	0.56	[0.23–1.36]	− 1.28	0.201
	Use of antibiotics	No	33.46	[4.57–245.05]	3.46	0.001*

* means a statistical significance (*P*<0.05)

### Step 4: estimation on the direct economic disease burden attributable to HAIs

In order to accurately estimate the direct economic disease burden attributable to HAIs, the approach adopted involves a 1:1 matching method to compare medical expenditures and hospitalization durations between hospitalized patients with and without HAIs. This method focuses on various medical expenditures including total medical expenditure, medicine expenditure, and antibiotics expenditure, as well as hospitalization days. These measures comprehensively reflect the economic burden and resource consumption associated with HAIs during hospitalization. By providing a detailed view of the additional costs incurred by hospitalized patients with HAIs, these measures highlight the financial impact on the healthcare system. The following specific steps were implemented in this TB hospital, as illustrated in Fig. 1:

1. Prior to the data analysis, we adjusted the medical expenditure data from 2018 to reflect 2019 values using the Consumer Price Indices (CPI) for medicines and healthcare services in Hubei Province [15]. The adjustment formula is expressed as follows: $E_{{Y_{{\text{b}}} }} { = }E_{{{\text{Y}}_{{\text{a}}} }} {\text{(1 + n}}_{{Y_{{{\text{a}} + 1}} }} {\text{\% )( 1 + n}}_{{Y_{{{\text{a}} + 2}} }} {)} \cdots {\text{ (1 + n}}_{{Y_{{\text{b}}} }} {\text{\% )}}$. Specifically, *E* represents the medical expenditures; *Y* denotes the year; and n indicates the annual increase in CPI.
2. Fig. 2 displays the respective numbers of TB hospitalized patients diagnosed with HAIs in the years 2018 and 2019, which were 44 and 34, respectively. Additional file 1: Table S3 provides an overview of the medical expenditures and hospitalization days for TB hospitalized patients with HAIs. Given that the data on medical expenditures and hospitalization days were skewed, the median, interquartile range (IQR), and the overall range (minimum to maximum values) were used to present the average levels of these variables. The average total medical expenditure increased from ¥30,730.70 in 2018 to ¥37,669.07 in 2019. Similarly, the average medicine expenditure rose by 77.83%, while the average hospitalization days increased slightly. The range of antibiotics expenditure broadened considerably, despite the average costs remaining stable. These trends highlighted an upward shift in healthcare spending and resource utilization for TB hospitalized patients with HAIs over 2018 and 2019.
3. Propensity Score Matching (PSM) was utilized to select a balanced cohort of TB hospitalized patients with and without HAIs. PSM has been extensively applied in medical research to mitigate selection bias and estimate the effects of exposure in observational studies [16, 17]. It operates by matching two groups with similar propensity scores (PS), which represent the likelihood of a patient being exposed to HAIs based on predefined patient characteristics, with scores ranging from 0 to 1 [18, 19]. In this TB hospital, the Generalized Boosted Model (GBM) was used to generate the PS [20]. A PSM method employing a caliper of 0.25 standard deviations (SD) of the PS facilitated the 1:1 matching without replacement, thus achieving a balanced comparison between TB hospitalized patients with and without HAIs. Based on the third step, which focuses on identification of the risk factors associated with HAIs, the covariates included in the models were gender, age, and use of invasive procedures such as central venous catheter, urine tube intubation, arteriovenous cannula, endotracheal intubation, mechanical ventilation, drainage, and tracheostomy. The variables including longer than 15 hospitalization days and use of antibiotics were excluded, since the antibiotics expenditure and hospitalization days were selected as measures for estimating the additional direct disease burden attributable to HAIs. The codes for the PSM analysis are available in Additional file 2. The resulting matched pairs were 44 and 34 for the years 2018 and 2019, respectively. Additional file 1: Table S4 displays the comparisons of covariates between the two groups before and after performing PSM, confirming the effectiveness of the matching in balancing the covariates.
4. After selecting the balanced groups of TB hospitalized patients with and without HAIs, the Wilcoxon matched-pairs signed-rank test was conducted to compare differences in medical expenditures and hospitalization days, thus assessing the additional direct economic disease burden attributable to HAIs. The codes for this statistical test are also included in Additional file 2. Table 4 shows that in both years of 2018 and 2019, TB hospitalized with HAIs consistently incurred much higher medical expenditures across all categories compared to those without HAIs. In 2018, the additional total medical expenditure was ¥15,417.31, with similar disparities in medicine and antibiotics expenditures, at ¥5754.74 and ¥2421.63 respectively (*P* < 0.01). The trend continued in 2019, where the additional total medical expenditure increased to ¥26,978.70, indicating a rising cost burden associated with HAIs. The additional medicine and antibiotics expenditures also increased, rising to ¥10,595.32 and ¥2218.66, respectively. Hospitalization days also reflected significant disparities, with HAIs patients hospitalized for much longer periods. In 2018, the additional hospitalization days were 11.5 days, and this gap widened in 2019 to 21.5 days. These indicate that HAIs were associated with substantially higher medical expenditures and longer hospital stays, with these disparities growing from 2018 to 2019. This underscores the critical financial and operational impacts of HAIs on healthcare systems.
5. Rosenbaum bounds for robust test has been widely applied to assess the sensitivity to hidden bias in observational studies [21]. Specifically, it is used to quantify the impact of unobserved confounding factors after performing the PSM analysis. In this case study, the sensitivity parameter Gamma (Γ) ranged from 1 to 2, which represents the degree of departure from random assignment due to an unobserved confounding factor. The codes for calculating Rosenbaum bounds are attached in Additional file 2. As indicated in Table S5, for all measures in both years 2018 and 2019, even when Gamma (Γ) equaled to a large value, such as 2, the *P* values (Sig +) were still lower than 0.05. These results indicated that the analyses on the additional direct economic disease burden were robust to hidden biases.
6. Different matching methods including 1: 2, 1: 3, and 1: 4, when conducting the PSM analysis, were employed to conduct the sensitivity analysis in order to test the robustness of the results generated from 1: 1 matching method in this case study. Table S6 shows that the additional total medical expenditure per TB hospitalized patient was ¥22,784.37 using 1: 3 matching method in 2018. It had a highest level of differences at 47.78%, compared to the remaining matching methods and measures. The lowest level of differences was 0.87% for the additional medicine expenditure per TB hospitalized patient using 1: 2 matching method in the same year. Additionally, as indicated in Additional file 1: Table S7, the results of Rosenbaum bounds for robust test showed that for different matching methods, the analyses on the additional direct economic disease burden for all measures were still robust to hidden biases.

**Table 4:** Additional direct economic disease burden attributable to HAIs from 2018 to 2019 (¥)

Year	Measures/per patient	TB hospitalized patients with HAIs	TB hospitalized patients without HAIs	Differences	*Z*	*P*
		Median (Q_25_, Q_75_)				
2018	Total medical expenditure	30,730.70 (18,438.59–59,474.81)	10,277.28 (7,028.36–21,898.79)	15,417.31 (6633.08–41,634.12)	4.77	< 0.01*
	Medicine expenditure	9181.29 (5467.41–16,634.13)	2450.13 (1375.44–5750.82)	5754.74 (1188.66–11,963.25)	4.93	< 0.01*
	Antibiotics expenditure	2902.72 (2125.28–5584.55)	923.84 (0.00–2111.95)	2421.63 (607.97–4768.49)	4.06	< 0.01*
	Hospitalization days	25 (14.5–34)	11.5 (6–18.5)	11.5 (4–22)	4.83	< 0.01*
2019	Total medical expenditure	37,669.07 (19,591.27–62,437.21)	8906.16 (5687.36–25,609.32)	26,978.70 (8637.42–55,782.94)	5.05	< 0.01*
	Medicine expenditure	16,326.64 (9363.05–28,571.62)	2340.40 (1015.68–5564.57)	10,595.32 (5030.52–26,701.55)	4.88	< 0.01*
	Antibiotics expenditure	2878.10 (2003.60–8020.37)	365.88 (0.00–1832.00)	2218.66 (1215.24–7711.61)	4.49	< 0.01*
	Hospitalization days	28.5 (18–40)	9.5 (3–13)	21.5 (8–38)	4.92	< 0.01*

* means a statistical significance (*P*<0.05)

## Possible applications

First, the application of this framework could be applied in single hospitals at the local level. Results generated from this framework could be applied for internal hospital management, providing critical insights for hospital managers. These generated results directly help hospital managers clearly understand the specific areas at high risk for HAIs, enabling targeted interventions, such as enhanced hand hygiene protocols, environmental cleaning, and disinfection procedures, as well as improved sterilization of medical equipment. This data-driven approach allows for the refinement of IPC measures, including isolation protocols for infected patients and antimicrobial stewardship programs, thereby improving patient safety and reducing the incidence of HAIs. Besides, by quantifying the economic burden attributable to HAIs, hospital managers can better allocate resources to areas that yield the highest return on investment in terms of infection prevention and patient care. Besides, integration of this framework into HIS could enable dynamic tracking and monitoring of HAIs, facilitating timely interventions, such as outbreak response and continuous staff training on IPC practices. Particularly, if such framework could be implemented in an established alliance of hospitals, it is able to more effectively consolidate and share critical data, just as the function of real-time surveillance of existing HAIs systems [22, 23]. Additionally, optimizing resources allocation and tailoring IPC measures could be significantly enhanced based on the insights provided by this framework, such as the deployment of rapid diagnostic tests and the use of personal protective equipment.

Second, the application of this framework could be applied at the regional and national levels. Results generated from this framework can also be used for hospital governance, particularly for benchmarking across multiple hospitals [24, 25]. Benchmarking can enable hospitals to determine the most cost-effective IPC measures and share best practices by comparing their values. For example, if one of the compared hospitals exhibits a significantly lower direct economic disease burden attributable to HAIs compared to others, it can be identified as a model of best practice. Hospital managers and policy decision-makers can then figure out the measures or management mechanism formulated by the best practice, and promote them in a broader way to enhance healthcare outcomes. Such applications of benchmarking can improve patient safety and reduce the economic impact of HAIs, thereby benefiting the broader society. Additionally, results generated from this framework can be applied for inter-organizational learning [26]. Hospitals can use these results as basis of training or intervention programs to improve the awareness of infection control among health professionals. Health professionals can learn the latest updates about theory, methodology, and technology on controlling HAIs via regular workshops, training sessions, and feedback meetings, so that they can enhance their own capacity to implement relevant measures. This needs the involvement of third-party agencies to provide professional evaluation of hospital performance [27]. These agencies can perform regular monitoring, ensuring compliance with established IPC standards and effectiveness of implemented strategies.

Third, at the international level, the adaptability of this framework allows for its potential applications in other countries, especially in settings with limited resources. More empirical evidence and experience could be generated to help local hospitals enhance the efficiency of infection control strategies, thereby reducing the attributable medical expenditures and improving quality of health. Moreover, this framework can be continuously refined and adjusted through its application across various international countries, leading to advancements in global health standards and policies. This can also be implemented by non-profit organizations, such as WHO. This collaborative international approach could address the global challenge of HAIs effectively and enhance the resilience and responsiveness of health systems around the world, as supported by the WHO recent reports on global IPC initiatives [3].

## Challenges to implement this framework

First, the challenge to implement this framework is quality of data extracted from hospitals. Data extraction is highly related to the HIS capabilities within each hospital, since data for different variables are sourced from different subsystems of the HIS. Inconsistencies in data management and integration across these subsystems can lead to inaccuracies that compromise the reliability of the framework. The variance in technological infrastructure between hospitals, especially in lower-resource settings, can pose significant challenges as well [28]. Moreover, not all hospitals are equipped with advanced HIS systems that can provide the detailed and accurate data necessary for effective application of the framework. This technological disparity can result in significant differences in data quality and accessibility, complicating the implementation process and potentially skewing results.

Second, the adoption of this framework in other countries need to navigate varying levels of regulatory compliance, data privacy standards, and government support. For instance, data protection regulations in some countries may restrict the types of data that can be collected and how it can be used, which may limit the framework’s applicability and effectiveness [29]. Cultural differences in the management and operation of hospitals can also influence the consistency and completeness of data collection. Training and capacity building are also crucial for successful implementation of this framework. Hospital staff need to be trained in how to use the system and in understanding the importance of accurate data entry. Without proper training and a clear understanding of the framework’s objectives, the risk of data entry errors increases, which could compromise data quality and the subsequent analyses.

Third, the special feature of TB may pose challenges to implement this framework. For example, since the medical expenditures for hospitalized patients with MDR-TB are usually significantly higher than those for hospitalized patients with single-drug-resistant TB (SDR-TB), when conducting the PSM analysis, the covariate should include the status whether a hospitalized patient has MDR-TB or SDR-TB. This ensures that the PSM analysis can accurately account for the medical expenditures associated with different types of TB resistance. Besides, for TB hospitalized patients, use of antibiotics is a common treatment. The intensity of antibiotics use, the type of antibiotics, and whether they are used reasonably can all be possible aspects to consider. Although in our systematic review, we identified several risk factors for HAIs, it is essential to continually consult relevant experts/doctors and review the latest literature to ensure that all significant TB-specific risk factors are included as covariates in the PSM analysis. Thus, the analysis can more precisely attribute the estimated direct economic disease burden solely to HAIs. This can be also applied to other communicable diseases or special diseases. While this case study only focused on the burden of direct medical expenditures, the burden of direct non-medical expenses, such as transportation, caregiver time, and lost income, is also significant. Future research should incorporate these non-medical costs to provide a more comprehensive assessment of the economic burden of HAIs. Patient surveys and cost diaries can be futher applied to capture these additional expenses.

## Supplementary Information

**Additional file 1: Table S1**. Inclusion and exclusion criteria for TB hospitalized patients with and without HAIs. **Table S2**. Description and assigned values of the potential risk factors associated with HAIs among TB hospitalized patients. **Table S3**. Summaries of medical expenditures and hospitalization days among TB hospitalized patients with HAIs from 2018 to 2019. **Table S4.** Comparisons of covariates between TB hospitalized patients with and without HAIs before and after performing PSM. **Table S5**. Rosenbaum bounds for robust test on the additional direct economic disease burden attributable to HAIs in 2018 and 2019. **Table S6**. Sensitivity analysis using different matching methods in 2018 and 2019. **Table S7.** Rosenbaum bounds for robust test across different PSM matching methods in 2018 and 2019.

**Additional file 2:** Codes for conducting univariate analysis in STATA software. Codes for conducting multiple logistic regression analysis in STATA software. Codes for performing PSM analysis in STATA software. Codes for conducting Wilcoxon matched-pairs signed-rank tests in STATA software. Codes for conducting Rosenbaum bounds for robust test in STATA software.

## Data Availability

Data and materials are accessible upon the reasonable request to the research team.

## Abbreviations

AMR: Antimicrobial resistance
CPI: Consumer Price Indices
GBM: Generalized Boosted Model
HAIs: Healthcare-associated infections
HICs: High-income countries
HIS: Hospital information system
IPC: Infection and prevention control
IQR: Interquartile range
LMICs: Low- and middle-income countries
MDR-TB: Multi-drug-resistant tuberculosis
OR: Odd ratio
PS: Propensity score
PSM: Propensity Score Matching
SD: Standard deviations
SDR-TB: Single-drug-resistant tuberculosis
TB: Tuberculosis
WHO: World Health Organization
