# Supplementary material for: Developing a framework for identifying risk factors and estimating direct economic disease burden attributable to healthcare-associated infections: a case study of a Chinese Tuberculosis hospital
Source: Glob Health Res Policy. 2024 Sep 9;9:33. doi: 10.1186/s41256-024-00375-w (PMC11382460; doi:10.1186/s41256-024-00375-w)
Supplement: Supplementary file 1 — Additional file 1: Table S1. Inclusion and exclusion criteria for TB hospitalized patients with and without HAIs. Table S2. Description and assigned values of the potential risk factors associated with HAIs among TB hospitalized patients. Table S3. Summaries of medical expenditures and hospitalization days among TB hospitalized patients with HAIs from 2018 to 2019. Table S4. Comparisons of covariates between TB hospitalized patients with and without HAIs before and after performing PSM. Table S5. Rosenbaum bounds for robust test on the additional direct economic disease burden attributable to HAIs in 2018 and 2019. Table S6. Sensitivity analysis using different matching methods in 2018 and 2019. Table S7. Rosenbaum bounds for robust test across different PSM matching methods in 2018 and 2019. [file 41256_2024_375_MOESM1_ESM.docx]

**Supplementary material 1**

**Table S1 Inclusion and exclusion criteria for TB hospitalized patients with and without HAIs**

| **Criteria** | **TB hospitalized patients with HAIs** | **TB hospitalized patients without HAIs** |
| --- | --- | --- |
| Inclusion criteria | 1. Patients were admitted to the hospital more than 48 hours, since HAIs were defined as infections were acquired by patients admitted to a hospital or other health care facility more than 48 hours and were not present or incubating at the time of admission but could appear after discharge, usually after 30 days. 2. Patients had completed necessary information regarding demographic and sociological information, mainly including gender and age, which had an impact on the occurrence of HAIs. | |
|  | Patients were clinically or etiologically diagnosed as HAIs. It means that positive findings of microbiology culture test were found from the microbiology laboratory. | Negative findings of microbiology culture test were found from the microbiology laboratory. |
| Exclusion criteria | 1. The period of hospitalization was less than 48 hours. 2. Patients did not have full necessary demographic and sociological information. 3. Patients had any other kind of infections at the time of admission. | |
|  | Negative findings of microbiological culture test were found from the microbiology laboratory. | Patients were diagnosed as HAIs. |

**Table S2 Description and assigned values of the potential risk factors associated with HAIs among TB hospitalized patients**

| **No** | **Potential risk factors** | **Description and assigned values** |
| --- | --- | --- |
| 1 | Gender | Male=1; Female=0 |
| 2 | Age group | Age > 60 years=1; Age ≤ 60years=0 |
| 3 | Invasive procedures | Yes(central venous catheter, urine tube intubation, arteriovenous cannula, endotracheal intubation, mechanical ventilation, drainage, and tracheostomy)=1; No=0 |
| 4 | Use of antibiotics | Yes=1; No=0 |
| 5 | Diabetes mellitus | Yes=1; No=0 |
| 6 | Length of hospitalization | Hospitalization days >15 days=1; Hospitalization days ≤15 days=0 |
| 7 | Underlying disease | Yes=1(hypertension, coronary heart disease, hepatopathy, renal failure, heart failure, and chronic obstructive pulmonary disease-COPD); No=0 |

**Table S3 Summaries of medical expenditures and hospitalization days among TB hospitalized patients with HAIs from 2018 to 2019 (¥)**

| **Year** | **Measures/per patient** | **Median** | **(Q_25_, Q_75_)** | **Minimum** | **Maximum** |
| --- | --- | --- | --- | --- | --- |
| 2018 | Total medical expenditure | 30,730.70 | (18,438.59-59,474.81) | 8,383.36 | 220,669.11 |
|  | Medicine expenditure | 9,181.29 | (5,467.41-16,634.13) | 2,036.42 | 67,750.38 |
|  | Antibiotics expenditure | 2,902.72 | (2,125.28-5,584.55) | 0.00 | 28,899.39 |
|  | Hospitalization days | 25 | (14.5, 34) | 11 | 134 |
| 2019 | Total medical expenditure | 37,669.07 | (19,591.27-62,437.21) | 10,863.69 | 170,969.33 |
|  | Medicine expenditure | 16,326.64 | (9,363.05-28,571.62) | 1,783.23 | 68,400.89 |
|  | Antibiotics expenditure | 2,878.10 | (2,003.60-8,020.37) | 0.00 | 22,028.23 |
|  | Hospitalization days | 28.5 | (18, 40) | 8 | 81 |

**Table S4 Comparisons of covariates between TB hospitalized patients with and without HAIs before and after performing PSM**

| **PSM** | **Year** | **Covariates** | | **TB hospitalized patients with HAIs** | **TB hospitalized patients without HAIs** | ***χ^2^***/t | ***P*** |
| --- | --- | --- | --- | --- | --- | --- | --- |
|  |  |  |  | **N (%)/**$\bar{\mathbf{x}}\boldsymbol{\pm SD}$ | |  |  |
| Before | 2018 | Gender | Male | 40 (90.91) | 6,413 (62.82) | 14.82 | <0.01* |
|  |  |  | Female | 4 (9.09) | 3,796 (37.18) |  |  |
|  |  | Age | | 51±20 | 45±19 | 1.84 | 0.073 |
|  |  | Invasive procedure | Yes | 12 (27.27) | 492 (4.82) | - | <0.01* |
|  |  |  | No | 32 (72.73) | 9,717 (95.18) |  |  |
|  | 2019 | Gender | Male | 27 (79.41) | 6,760 (62.24) | 4.26 | 0.039* |
|  |  |  | Female | 7 (20.59) | 4,101 (37.76) |  |  |
|  |  | Age | | 50±4 | 45±0.2 | 1.64 | 0.102 |
|  |  | Invasive procedure | Yes | 7 (20.59) | 659 (6.07) | - | 0.004* |
|  |  |  | No | 27 (79.41) | 10,202 (93.93) |  |  |
| After | 2018 | Gender | Male | 40 (90.91) | 40 (90.91) | - | 1.000 |
|  |  |  | Female | 4 (9.09) | 4 (9.09) |  |  |
|  |  | Age | | 51±20 | 50±21 | 0.05 | 0.959 |
|  |  | Invasive procedure | Yes | 12 (27.27) | 12 (27.27) | - | 1.000 |
|  |  |  | No | 32 (72.73) | 32 (72.73) |  |  |
|  | 2019 | Gender | Male | 27 (79.41) | 27 (79.41) | - | 1.000 |
|  |  |  | Female | 7 (20.59) | 7 (20.59) |  |  |
|  |  | Age | | 50±4 | 51±4 | 0.05 | 0.963 |
|  |  | Invasive procedure | Yes | 7 (20.59) | 7 (20.59) | - | 1.000 |
|  |  |  | No | 27 (79.41) | 27 (79.41) |  |  |

**Table S5 Rosenbaum bounds for robust test on the additional direct economic disease burden attributable to HAIs in 2018 and 2019**

| **Year** | **Measures/per patient** | **Gamma (Γ)** | **Sig-** | **Sig+** | **t-hat-** | **t-hat+** | **CI-** | **CI+** |
| --- | --- | --- | --- | --- | --- | --- | --- | --- |
| 2018 | Total medical expenditure | 1 | 9.1*10^-7^ | 9.1*10^-7^ | 22,142.1 | 22,142.1 | 13,072.8 | 36,031.0 |
|  |  | 1.2 | 5.2*10^-8^ | 1.0*10^-5^ | 18,955.6 | 25,148.2 | 10,046.3 | 42,306.0 |
|  |  | 1.5 | 7.2*10^-10^ | 0.000111 | 16,603.5 | 29,007.7 | 8,271.6 | 50,302.7 |
|  |  | 2 | 6.0*10^-13^ | 0.001263 | 13,239.9 | 35,591.1 | 6,211.0 | 58,232.2 |
|  | Medicine expenditure | 1 | 4.2*10^-7^ | 4.2*10^-7^ | 6,713.2 | 6,713.2 | 4,078.4 | 10,884.4 |
|  |  | 1.2 | 2.2*10^-8^ | 5.0*10^-6^ | 5,970.6 | 7,464.0 | 3,615.7 | 14,056.7 |
|  |  | 1.5 | 2.7*10^-10^ | 0.00006 | 5,171.6 | 8,691.2 | 2,800.5 | 17,944.0 |
|  |  | 2 | 1.9*10^-13^ | 0.000733 | 4,104.54 | 10,686.0 | 1,955.5 | 21,470.3 |
|  | Antibiotics expenditure | 1 | 0.000024 | 0.000024 | 2,451.0 | 2,451.0 | 1,491.6 | 3,647.3 |
|  |  | 1.2 | 2.1*10^-6^ | 0.000192 | 2,186.4 | 2,756.6 | 1,240.3 | 4,026.9 |
|  |  | 1.5 | 5.1*10^-8^ | 0.001509 | 1,917.6 | 3,094.7 | 813.6 | 4,465.2 |
|  |  | 2 | 1.1*10^-10^ | 0.01175 | 1,510.5 | 3,625.1 | 184.1 | 5,154.9 |
|  | Hospitalization days | 1 | 6.7*10^-7^ | 6.7*10^-7^ | 13.5 | 13.5 | 9.0 | 18.5 |
| **Year** | **Measures/per patient** | **Gamma (Γ)** | **Sig-** | **Sig+** | **t-hat-** | **t-hat+** | **CI-** | **CI+** |
| 2018 | Hospitalization days | 1.2 | 3.7*10^-8^ | 7.6*10^-6^ | 12.5 | 15.0 | 8.0 | 20.5 |
|  |  | 1.5 | 4.9*10^-10^ | 0.000087 | 11.0 | 16.5 | 6.5 | 23.0 |
|  |  | 2 | 3.7*10^-13^ | 0.001023 | 9.0 | 18.5 | 4.5 | 26.5 |
| 2019 | Total medical expenditure | 1 | 8.1*10^-7^ | 8.1*10^-7^ | 29,385.7 | 29,385.7 | 19,672.8 | 44,246.1 |
|  |  | 1.2 | 6.5*10^-8^ | 6.8*10^-6^ | 27,187.3 | 32,313.7 | 17,554.6 | 47,987.9 |
|  |  | 1.5 | 1.5*10^-9^ | 0.000058 | 24,110.5 | 36,455.5 | 14,932.0 | 53,779.9 |
|  |  | 2 | 2.9*10^-12^ | 0.000504 | 20,882.3 | 41,439.8 | 11,636.1 | 59,942.3 |
|  | Medicine expenditure | 1 | 1.8*10^-7^ | 1.8*10^-7^ | 16,441.6 | 16,441.6 | 10,422.9 | 23,262.7 |
|  |  | 1.2 | 1.3*10^-8^ | 1.7*10^-6^ | 15,091.1 | 18,321.3 | 9,248.1 | 24,889.7 |
|  |  | 1.5 | 2.3*10^-10^ | 0.000016 | 13,549.5 | 20,338.3 | 7,933.4 | 27,006.4 |
|  |  | 2 | 3.2*10^-13^ | 0.000161 | 11,407.8 | 22,450.7 | 6,649.5 | 30,074.5 |
|  | Antibiotics expenditure | 1 | 3.3*10^-7^ | 3.3*10^-7^ | 3,828.4 | 3,828.4 | 2,139.6 | 5,697.9 |
|  |  | 1.2 | 2.4*10^-8^ | 2.9*10^-6^ | 3,339.2 | 4,454.2 | 1,998.0 | 6,724.4 |
|  |  | 1.5 | 4.8*10^-10^ | 0.000027 | 2,742.4 | 4,852.7 | 1,671.7 | 8,249.3 |
|  |  | 2 | 7.6*10^-13^ | 0.000249 | 2,305.6 | 5,439.6 | 1,362.4 | 9,327.6 |
|  | Hospitalization days | 1 | 6.2*10^7^ | 6.2*10^7^ | 20.0 | 20.0 | 14.0 | 26.5 |
|  |  | 1.2 | 4.9*10^-8^ | 5.3*10^-6^ | 18.5 | 21.5 | 12.5 | 28.5 |
|  |  | 1.5 | 1.1*10^-9^ | 0.000046 | 17.0 | 23.0 | 11.0 | 30.5 |
|  |  | 2 | 2.0*10^-12^ | 0.000409 | 15.0 | 25.5 | 8.5 | 34.0 |

**Note:** gamma: log odds of differential assignment due to unobserved confounding factors; sig+: upper bound significance level; sig-: lower bound significance level; t-hat+: upper bound Hodges-Lehmann point estimate; t-hat-: lower bound Hodges-Lehmann point estimate; CI+: upper bound confidence interval (α=0.95); CI-: lower bound confidence interval (α=0.95).

**Table S6 Sensitivity analysis using different matching methods in 2018 and 2019**

| **Year** | **Matching methods** | **Measures/per patient** | **TB hospitalized patients with HAIs** | **TB hospitalized patients without HAIs** | **Differences** | ***Z*** | ***P*** | **Level of differences (%)** |
| --- | --- | --- | --- | --- | --- | --- | --- | --- |
|  |  |  | **Median (Q_25_, Q_75_)** | |  |  |  |  |
| 2018 | 1 : 2 | Total medical expenditure | 30,730.70 (18,438.59-59,474.81) | 11,030.67 (7,922.57-17,471.01) | 20,399.14 (7,328.92-49,188.88) | 5.45 | <0.01* | 32.31 |
|  |  | Medicine expenditure | 9,181.29 (5,467.41-16,634.13) | 2,859.83 (1,808.45-4,844.92) | 5,805.02 (2,071.23-13,853.10) | 5.52 | <0.01* | 0.87 |
|  |  | Antibiotics expenditure | 2,902.72 (2,125.28-5,584.55) | 1,071.42 (515.49-1,405.87) | 2,111.11 (790.46-5,251.86) | 4.87 | <0.01* | 12.82 |
|  |  | Hospitalization days | 25 (14.5-34) | 11.5 (7.5-14.8) | 13.8 (5.5-19.8) | 5.52 | <0.01* | 20.00 |
|  | 1 : 3 | Total medical expenditure | 30,730.70 (18,438.59-59,474.81) | 9,676.63 (7,685.60-16,875.56) | 22,784.37 (9,590.91-42,720.40) | 5.28 | <0.01* | 47.78 |
|  |  | Medicine expenditure | 9,181.29 (5,467.41-16,634.13) | 2,136.67 (1,255.79-4,941.48) | 6,640.56 (3,276.39-13,700.33) | 5.49 | <0.01* | 15.39 |
|  |  | Antibiotics expenditure | 2,902.72 (2,125.28-5,584.55) | 727.02 (0.00-1,262.74) | 2,760.39 (1,269.59-4,897.37) | 5.01 | <0.01* | 13.99 |
|  |  | Hospitalization days | 25 (14.5-34) | 9 (9-12) | 15 (8-22) | 5.64 | <0.01* | 30.43 |
|  | 1 : 4 | Total medical expenditure | 30,730.70 (18,438.59-59,474.81) | 9,585.57 (7,678.01-19,592.38) | 20,243.71 (8,747.38-46,875.27) | 5.12 | <0.01* | 31.31 |
|  |  | Medicine expenditure | 9,181.29 (5,467.41-16,634.13) | 2,061.53 (1,529.85-3,974.07) | 6,659.80 (3,563.32-14,082.98) | 5.40 | <0.01* | 15.73 |
|  |  | Antibiotics expenditure | 2,902.72 (2,125.28-5,584.55) | 724.32 (461.06-1,359.02) | 2,637.87 (1,210.61-5,025.85) | 5.22 | <0.01* | 8.93 |
|  |  | Hospitalization days | 25 (14.5-34) | 10.3 (7.8-13.8) | 13.8 (6.5-21.3) | 5.75 | <0.01* | 20.00 |
| 2019 | 1 : 2 | Total medical expenditure | 37,669.07 (19,591.27-62,437.21) | 8,750.25 (8,750.25-8,750.25) | 25,370.96 (10,128.70-52,404.16) | 4.78 | <0.01* | 5.96 |
|  |  | Medicine expenditure | 16,326.64 (9,363.05-28,571.62) | 2,754.39 (2,754.39-2,754.39) | 11,220.11 (5,410.39-27,298.29) | 5.07 | <0.01* | 9.56 |
|  |  | Antibiotics expenditure | 2,878.10 (2,003.60-8,020.37) | 1,073.54 (1,073.54-1,073.54) | 1,879.65 (982.34-7,488.33) | 4.86 | <0.01* | 15.28 |
|  |  | Hospitalization days | 28.5 (18-40) | 10.5 (10.5-10.5) | 17.5 (0.5-17.0) | 4.97 | <0.01* | 18.60 |
|  | 1 : 3 | Total medical expenditure | 37,669.07 (19,591.27-62,437.21) | 10,685.05 (10,685.05-10,685.05) | 27,837.71 (8,625.95-50,469.35) | 5.09 | <0.01* | 3.18 |
|  |  | Medicine expenditure | 16,326.64 (9,363.05-28,571.62) | 2,984.59 (2,984.59-2,984.59) | 12,292.67 (5,180.19-26,325.93) | 5.07 | <0.01* | 11.61 |
|  |  | Antibiotics expenditure | 2,878.10 (2,003.60-8,020.37) | 1,285.16 (1,285.16-1,285.16) | 1,668.03 (776.38-7,276.71) | 4.76 | <0.01* | 24.82 |
|  |  | Hospitalization days | 28.5 (18-40) | 15 (15-15) | 14 (4-25) | 4.67 | <0.01* | 34.88 |
| **Year** | **Matching methods** | **Measures/per patient** | **TB hospitalized patients with HAIs** | **TB hospitalized patients without HAIs** | **Differences** | ***Z*** | ***P*** | **Level of differences (%)** |
|  |  |  | **Median (Q_25_, Q_75_)** | |  |  |  |  |
| 2019 | 1 : 4 | Total medical expenditure | 37,669.07 (19,591.27-62,437.21) | 8,750.25 (8,750.25-8,750.25) | 29,371.52 (10,560.76-49280.06) | 5.09 | <0.01* | 8.67 |
|  |  | Medicine expenditure | 16,326.64 (9,363.05-28,571.62) | 2,754.39 (2,754.39-2,754.39) | 12,522.87 (5,410.39-25,002.25) | 5.07 | <0.01* | 5.57 |
|  |  | Antibiotics expenditure | 2,878.10 (2,003.60-8,020.37) | 1,073.54 (1,073.54-1,073.54) | 1,885.41 (982.34-7,488.33) | 4.86 | <0.01* | 15.02 |
|  |  | Hospitalization days | 28.5 (18-40) | 10.5 (10.5-10.5) | 18 (7.5-29.5) | 4.88 | <0.01* | 16.28 |

**Note:** The ‘level of differences’ was calculated by determining the differences between the differences for each outcome using different matching methods, including 1 : 2, 1 : 3, and 1 : 4, and the differences for each outcome using 1 : 1 matching method. These differences were then divided by the differences for each outcome using 1 : 1 matching method, which were presented as percentages.

**Table S7 Rosenbaum bounds for robust test across different PSM matching methods in 2018 and 2019**

| **Year** | **Matching methods** | **Measures/per patient** | **Gamma (Γ)** | **Sig-** | **Sig+** | **t-hat-** | **t-hat+** | **CI-** | **CI+** |
| --- | --- | --- | --- | --- | --- | --- | --- | --- | --- |
| 2018 | 1 : 2 | Total medical expenditure | 1 | 1.9*10^-8^ | 1.9*10^-8^ | 24,647.9 | 24,647.9 | 16,233.5 | 41,830.7 |
|  |  |  | 1.2 | 7.4*10^-10^ | 3.0*10^-7^ | 22,083.4 | 27,922.7 | 14,557.8 | 48,616.9 |
|  |  |  | 1.5 | 5.6*10^-12^ | 4.7*10^-6^ | 19,391.4 | 32,888.1 | 12,224.0 | 54,673.1 |
|  |  |  | 2 | 1.8*10^-15^ | 0.000076 | 16,276.3 | 41,280.3 | 9,646.9 | 62,860.7 |
|  |  | Medicine expenditure | 1 | 1.3*10^-8^ | 1.3*10^-8^ | 7,650.2 | 7,650.2 | 5,099.0 | 11,662.5 |
|  |  |  | 1.2 | 4.8*10^-10^ | 2.1*10^-7^ | 7,024.2 | 8,401.3 | 4,542.4 | 14,399.7 |
|  |  |  | 1.5 | 3.4*10^-12^ | 3.4*10^-6^ | 6,068.8 | 9,372.0 | 3,754.8 | 20,137.0 |
|  |  |  | 2 | 1.0*10^-15^ | 0.000056 | 5,108.1 | 11,535.6 | 2,980.3 | 24,528.6 |
|  |  | Antibiotics expenditure | 1 | 6.0*10^-7^ | 6.0*10^-7^ | 2,654.6 | 2,654.6 | 1,715.5 | 3,774.4 |
|  |  |  | 1.2 | 3.3*10^-8^ | 6.9*10^-6^ | 2,377.7 | 2,956.3 | 1,465.0 | 4,165.4 |
|  |  |  | 1.5 | 4.3*10^-10^ | 0.00008 | 2,060.4 | 3,247.6 | 1,116.5 | 4,598.2 |
|  |  |  | 2 | 3.2*10^-13^ | 0.000945 | 1,738.0 | 3,745.9 | 771.6 | 5,464.7 |
|  |  | Hospitalization days | 1 | 9.9*10^-9^ | 9.9*10^-9^ | 13.5 | 13.5 | 9.8 | 18.5 |
|  |  |  | 1.2 | 3.5*10^-10^ | 1.6*10^-7^ | 12.5 | 14.5 | 8.8 | 20.8 |
|  |  |  | 1.5 | 2.4*10^-12^ | 2.7*10^-6^ | 11.3 | 16.3 | 7.8 | 23.5 |
|  |  |  | 2 | 6.7*10^-16^ | 0.000046 | 10.0 | 18.5 | 6.3 | 27.8 |
|  | 1 : 3 | Total medical expenditure | 1 | 4.8*10^-8^ | 4.8*10^-8^ | 24,176.2 | 24,176.2 | 16,380.6 | 40,923.7 |
|  |  |  | 1.2 | 2.0*10^-9^ | 6.9*10^-7^ | 21,941.7 | 27,249.1 | 14,630.4 | 48,532.1 |
|  |  |  | 1.5 | 1.8*10^-11^ | 0.00001 | 19,483.6 | 31,720.9 | 12,262.5 | 54,646.8 |
|  |  |  | 2 | 6.9*10^-15^ | 0.000151 | 16,620.4 | 40,550.7 | 9,895.9 | 62,661.7 |
|  |  | Medicine expenditure | 1 | 2.1*10^-8^ | 2.1*10^-8^ | 7,539.1 | 7,539.1 | 5,316.8 | 11,392.5 |
|  |  |  | 1.2 | 7.9*10^-10^ | 3.2*10^-7^ | 6,859.5 | 8,280.0 | 4,726.1 | 14,735.0 |
|  |  |  | 1.5 | 6.1*10^-12^ | 5.0*10^-6^ | 6,131.1 | 9,388.2 | 4,045.4 | 20,152.1 |
|  |  |  | 2 | 1.9*10^-15^ | 0.00008 | 5,320.1 | 11,344.0 | 3,250.0 | 24,745.4 |
|  |  | Antibiotics expenditure | 1 | 3.3*10^-7^ | 3.3*10^-7^ | 2,652.7 | 2,652.7 | 1,764.0 | 3,706.5 |
|  |  |  | 1.2 | 1.7*10^-8^ | 4.0*10^-6^ | 2,418.7 | 2,864.4 | 1,477.8 | 4,076.5 |
|  |  |  | 1.5 | 2.0*10^-10^ | 0.000049 | 2,100.4 | 3,243.3 | 1,191.6 | 4,582.5 |
|  |  |  | 2 | 1.3*10^-13^ | 0.000617 | 1,779.8 | 3,683.6 | 854.6 | 5,389.7 |
|  |  | Hospitalization days | 1 | 8.9*10^-9^ | 8.9*10^-9^ | 14.0 | 14.0 | 10.8 | 19.2 |
| **Year** | **Matching methods** | **Measures/per patient** | **Gamma (Γ)** | **Sig-** | **Sig+** | **t-hat-** | **t-hat+** | **CI-** | **CI+** |
| 2018 | 1 : 3 | Hospitalization days | 1.2 | 3.2*10^-10^ | 1.5*10^-7^ | 13.1 | 15.2 | 10.0 | 21.8 |
|  |  |  | 1.5 | 2.2*10^-12^ | 2.5*10^-6^ | 12.2 | 16.5 | 9.0 | 24.7 |
|  |  |  | 2 | 5.6*10^-16^ | 0.000042 | 11.0 | 19.1 | 7.8 | 29.0 |
|  | 1 : 4 | Total medical expenditure | 1 | 5.8*10^-8^ | 5.8*10^-8^ | 24,689.6 | 24,689.6 | 16,250.0 | 40,250.7 |
|  |  |  | 1.2 | 2.5*10^-9^ | 8.2*10^-7^ | 21,680.5 | 27,144.4 | 13,826.9 | 46,068.3 |
|  |  |  | 1.5 | 2.2*10^-11^ | 0.000012 | 19,174.0 | 31,299.2 | 11,639.5 | 52,475.1 |
|  |  |  | 2 | 9.1*10^-15^ | 0.000174 | 16,287.3 | 40,147.6 | 8,928.5 | 61,633.0 |
|  |  | Medicine expenditure | 1 | 2.4*10^-8^ | 2.4*10^-8^ | 7,638.9 | 7,638.9 | 5,301.6 | 11,487.9 |
|  |  |  | 1.2 | 9.2*10^-10^ | 3.6*10^-7^ | 6,977.5 | 8,314.2 | 4,708.1 | 14,718.6 |
|  |  |  | 1.5 | 7.2*10^-12^ | 5.5*10^-6^ | 6,195.9 | 9,438.4 | 4,104.1 | 19,627.5 |
|  |  |  | 2 | 2.3*10^-15^ | 0.000088 | 5,339.2 | 11,403.8 | 3,205.3 | 24,690.7 |
|  |  | Antibiotics expenditure | 1 | 2.9*10^-7^ | 2.9*10^-7^ | 2,523.0 | 2,523.0 | 1,661.5 | 3,588.8 |
|  |  |  | 1.2 | 1.5*10^-8^ | 3.6*10^-6^ | 2,230.7 | 2,758.2 | 1,395.7 | 3,985.3 |
|  |  |  | 1.5 | 1.7*10^-10^ | 0.000045 | 1,996.4 | 3,114.4 | 1,145.9 | 4,548.5 |
|  |  |  | 2 | 1.1*10^-13^ | 0.000566 | 1,673.8 | 3,563.7 | 771.3 | 5,323.9 |
|  |  | Hospitalization days | 1 | 5.2*10^-9^ | 5.2*10^-9^ | 14.0 | 14.0 | 10.5 | 18.8 |
|  |  |  | 1.2 | 1.7*10^-10^ | 8.9*10^-8^ | 12.8 | 15.0 | 9.6 | 21.4 |
|  |  |  | 1.5 | 1.1*10^-12^ | 1.6*10^-6^ | 11.8 | 16.3 | 8.6 | 24.4 |
|  |  |  | 2 | 2.2*10^-16^ | 0.000028 | 10.5 | 18.8 | 7.4 | 28.1 |
| 2019 | 1 : 2 | Total medical expenditure | 1 | 8.8*10^-7^ | 8.8*10^-7^ | 28,904.7 | 28,904.7 | 18,461.0 | 44,275.3 |
|  |  |  | 1.2 | 7.1*10^-8^ | 7.3*10^-6^ | 25,566.3 | 32,263.5 | 16,644.4 | 47,770.0 |
|  |  |  | 1.5 | 1.7*10^-9^ | 0.000062 | 22,335.2 | 35,413.9 | 12,430.0 | 53,268.2 |
|  |  |  | 2 | 3.3*10^-12^ | 0.000538 | 19,611.3 | 41,866.1 | 10,011.3 | 59,585.2 |
|  |  | Medicine expenditure | 1 | 2.0*10^-7^ | 2.0*10^-7^ | 16,004.8 | 16,004.8 | 10,330.0 | 23,059.9 |
|  |  |  | 1.2 | 1.4*10^-8^ | 1.9*10^-6^ | 14,536.4 | 17,883.2 | 8,920.9 | 24,810.5 |
|  |  |  | 1.5 | 2.6*10^-10^ | 0.000018 | 12,909.9 | 19,463.3 | 7,702.6 | 26,631.0 |
|  |  |  | 2 | 3.6*10^-13^ | 0.000173 | 11,042.7 | 21,989.0 | 6,550.6 | 29,565.4 |
|  |  | Antibiotics expenditure | 1 | 5.8*10^-7^ | 5.8*10^-7^ | 3,426.4 | 3,426.4 | 1,516.6 | 5,260.2 |
|  |  |  | 1.2 | 4.4*10^-8^ | 4.9*10^-6^ | 2,711.5 | 4,007.8 | 1,351.6 | 6,366.0 |
|  |  |  | 1.5 | 9.7*10^-10^ | 0.000043 | 2,260.8 | 4,378.5 | 1,144.5 | 7,836.0 |
| **Year** | **Matching methods** | **Measures/per patient** | **Gamma (Γ)** | **Sig-** | **Sig+** | **t-hat-** | **t-hat+** | **CI-** | **CI+** |
| 2019 | 1 : 2 | Antibiotics expenditure | 2 | 1.7*10^-12^ | 0.000389 | 1,627.5 | 4,976.0 | 936.8 | 8,878.7 |
|  |  | Hospitalization days | 1 | 3.4*10^-7^ | 3.4*10^-7^ | 18.5 | 18.5 | 13.0 | 26.5 |
|  |  |  | 1.2 | 2.5*10^-8^ | 3.0*10^-6^ | 17.5 | 20.5 | 11.5 | 28.3 |
|  |  |  | 1.5 | 5.0*10^-10^ | 0.000028 | 15.5 | 22.8 | 10.0 | 31.0 |
|  |  |  | 2 | 7.9*10^-13^ | 0.00026 | 13.5 | 25.5 | 8.3 | 34.0 |
|  | 1 : 3 | Total medical expenditure | 1 | 4.4*10^-7^ | 4.4*10^-7^ | 28,719.3 | 28,719.3 | 18,068.5 | 42,120.2 |
|  |  |  | 1.2 | 3.3*10^-8^ | 3.9*10^-6^ | 25,622.0 | 31,097.6 | 16,434.2 | 46,068.0 |
|  |  |  | 1.5 | 7.0*10^-10^ | 0.000035 | 21,953.1 | 33,882.5 | 13,247.4 | 51,038.4 |
|  |  |  | 2 | 1.2*10^-12^ | 0.000319 | 19,258.1 | 38,239.6 | 9,353.3 | 58,155.4 |
|  |  | Medicine expenditure | 1 | 2.2*10^-7^ | 2.2*10^-7^ | 15,217.9 | 15,217.9 | 10,068.6 | 22,361.8 |
|  |  |  | 1.2 | 1.5*10^-8^ | 2.0*10^-6^ | 14,027.6 | 17,185.1 | 8,651.4 | 24,182.4 |
|  |  |  | 1.5 | 2.9*10^-10^ | 0.000019 | 12,572.9 | 18,765.2 | 7,488.6 | 26,078.3 |
|  |  |  | 2 | 4.1*10^-13^ | 0.000185 | 10,818.5 | 21,291.0 | 6,277.1 | 28,867.4 |
|  |  | Antibiotics expenditure | 1 | 8.1*10^-7^ | 8.1*10^-7^ | 3,384.7 | 3,384.7 | 1,405.6 | 5,135.8 |
|  |  |  | 1.2 | 6.5*10^-8^ | 6.8*10^-6^ | 2,780.4 | 3,985.7 | 1,281.7 | 6,237.2 |
|  |  |  | 1.5 | 1.5*10^-9^ | 0.000058 | 2,162.1 | 4,311.8 | 1,043.7 | 7,696.2 |
|  |  |  | 2 | 2.9*10^-12^ | 0.000504 | 1,655.3 | 4,852.9 | 855.7 | 8,738.6 |
|  |  | Hospitalization days | 1 | 1.2*10^-6^ | 1.2*10^-6^ | 16.3 | 16.3 | 10.7 | 23.8 |
|  |  |  | 1.2 | 9.8*10^-8^ | 9.6*10^-6^ | 14.8 | 18.2 | 9.3 | 25.9 |
|  |  |  | 1.5 | 2.4*10^-9^ | 0.000079 | 13.3 | 19.8 | 7.8 | 28.3 |
|  |  |  | 2 | 5.1*10^-12^ | 0.000671 | 11.3 | 22.8 | 5.7 | 31.8 |
|  | 1 : 4 | Total medical expenditure | 1 | 2.6*10^-7^ | 2.6*10^-7^ | 29,573.0 | 29,573.0 | 19,998.5 | 42,594.5 |
|  |  |  | 1.2 | 1.9*10^-8^ | 2.4*10^-6^ | 27,138.9 | 32,440.8 | 18,677.1 | 47,114.3 |
|  |  |  | 1.5 | 3.6*10^-10^ | 0.000022 | 23,592.8 | 35,032.0 | 15,617.8 | 51,997.8 |
|  |  |  | 2 | 5.4*10^-13^ | 0.000213 | 21,279.2 | 39,442.7 | 11,273.5 | 58,621.5 |
|  |  | Medicine expenditure | 1 | 2.0*10^-7^ | 2.0*10^-7^ | 15,404.6 | 15,404.6 | 10,510.5 | 22,948.3 |
|  |  |  | 1.2 | 1.4*10^-8^ | 1.9*10^-6^ | 14,380.6 | 17,642.6 | 9,310.4 | 24,904.2 |
|  |  |  | 1.5 | 2.6*10^-10^ | 0.000018 | 12670.7 | 19,235.1 | 8,109.1 | 26,724.7 |
|  |  |  | 2 | 3.6*10^-13^ | 0.000173 | 11184.6 | 21,993.0 | 6,997.3 | 29,654.1 |
|  |  | Antibiotics expenditure | 1 | 3.4*10^-7^ | 3.4*10^-7^ | 3404.6 | 3,404.6 | 1,709.1 | 5,353.2 |
|  |  |  | 1.2 | 2.5*10^-8^ | 3.1*10^-6^ | 2954.1 | 3,910.4 | 1,539.2 | 6,265.9 |
| **Year** | **Matching methods** | **Measures/per patient** | **Gamma (Γ)** | **Sig-** | **Sig+** | **t-hat-** | **t-hat+** | **CI-** | **CI+** |
| 2019 | 1 : 4 | Antibiotics expenditure | 1.5 | 5.1*10^-10^ | 0.000028 | 2,443.5 | 4,422.1 | 1,334.7 | 7,934.5 |
|  |  |  | 2 | 8.0*10^-13^ | 0.000261 | 1,883.7 | 4,900.0 | 1,115.8 | 9,042.0 |
|  |  | Hospitalization days | 1 | 6.2*10^-7^ | 6.2*10^-7^ | 18.6 | 18.6 | 12.8 | 26.1 |
|  |  |  | 1.2 | 4.9*10^-8^ | 5.3*10^-6^ | 16.8 | 20.3 | 11.3 | 28.3 |
|  |  |  | 1.5 | 1.1*10^-9^ | 0.000046 | 15.8 | 22.3 | 10.1 | 30.8 |
|  |  |  | 2 | 2.0*10^-12^ | 0.000414 | 13.8 | 25.3 | 7.8 | 33.6 |
