# Supplementary material for: Developing a framework for identifying risk factors and estimating direct economic disease burden attributable to healthcare-associated infections: a case study of a Chinese Tuberculosis hospital
Source: Glob Health Res Policy. 2024 Sep 9;9:33. doi: 10.1186/s41256-024-00375-w (PMC11382460; doi:10.1186/s41256-024-00375-w)
Supplement: Supplementary file 2 — Additional file 2: Codes for conducting univariate analysis in STATA software. Codes for conducting multiple logistic regression analysis in STATA software. Codes for performing PSM analysis in STATA software. Codes for conducting Wilcoxon matched-pairs signed-rank tests in STATA software. Codes for conducting Rosenbaum bounds for robust test in STATA software. [file 41256_2024_375_MOESM2_ESM.docx]

**Supplementary material 2**

**Codes for conducting univariate analysis in STATA software**

tabulate gender HAIs, chi2 exact expected

tabulate age HAIs, chi2 exact expected

tabulate IP HAIs, chi2 exact expected

tabulate H HAIs, chi2 exact expected

tabulate NS HAIs, chi2 exact expected

tabulate TR HAIs, chi2 exact expected

tabulate ST HAIs, chi2 exact expected

tabulate DM HAIs, chi2 exact expected

tabulate UD HAIs, chi2 exact expected

tabulate AN HAIs, chi2 exact expected

**Codes for conducting multiple logistic regression analysis in STATA software**

logistic HAIs gender age IP

logistic HAIs gender age H

logistic HAIs gender age NS

logistic HAIs gender age TR

logistic HAIs gender age ST

logistic HAIs gender age DM

logistic HAIs gender age UD

**Codes for performing PSM analysis in STATA software**

global treatment HAIs

global ylist TME ME AE H

global xlist age1 gender IP

program boost_plugin,plugin using("D:/Stata14MP-64/boost64.dll")

gen x=uniform()

sort x

set seed 1000

boost $treatment $xlist,dist(logistic) maxiter(1000) in pred(p) shrink(0.0005) bag(0.5) train(0.8) inter(5)

summarize p

#Data for 2018

#1:1

psmatch2 $treatment,out($ylist) pscore(p) caliper(0.00148705) noreplacement common logit descending

#1:2

psmatch2 $treatment,out($ylist) pscore(p) n(2) caliper(0.00148705) common logit

#1:3

psmatch2 $treatment,out($ylist) pscore(p) n(3) caliper(0.00148705) common logit

#1:4

psmatch2 $treatment,out($ylist) pscore(p) n(4) caliper(0.00148705) common logit

#Data for 2019

#1:1

psmatch2 $treatment,out($ylist) pscore(p) caliper(0.00001175) noreplacement common logit descending

#1:2

psmatch2 $treatment,out($ylist) pscore(p) n(2) caliper(0.00001175) common logit

#1:3

psmatch2 $treatment,out($ylist) pscore(p) n(3) caliper(0.00001175) common logit

#1:4

psmatch2 $treatment,out($ylist) pscore(p) n(4) caliper(0.00001175) common logit

sort _id

**Codes for conducting Wilcoxon matched-pairs signed-rank tests in STATA software**

signrank TME=TME1

signrank ME=ME1

signrank AE=AE1

signrank HD=HD1

**Codes for conducting Rosenbaum bounds for robust test in STATA software**

gen delta=TME-_TME if _treated==1 & _support==1

rbounds delta,gamma(1 1.2 1.5 2)

gen delta1=ME-_ME if _treated==1 & _support==1

rbounds delta1,gamma(1 1.2 1.5 2)

gen delta2=AE-_AE if _treated==1 & _support==1

rbounds delta2,gamma(1 1.2 1.5 2)

gen delta3=H-_H if _treated==1 & _support==1

rbounds delta3,gamma(1 1.2 1.5 2)
